# Carbon Nanotube Sheets/Elastomer Bilayer Harvesting Electrode with Biaxially Generated Electrical Energy

**DOI:** 10.3390/polym16172477

**Published:** 2024-08-30

**Authors:** Seongjae Oh, Hyeon Ji Kim, Seon Lee, Keon Jung Kim, Shi Hyeong Kim

**Affiliations:** 1 Department of Advanced Textile R&D, Korea Institute of Industrial Technology, Ansan 15588, Republic of Korea; 5tjdwo@kitech.re.kr (S.O.); khj111543@gmail.com (H.J.K.); dltjs99@kitech.re.kr (S.L.); 2 Semiconductor R&D Center, Samsung Electronics, Hwaseong 18448, Republic of Korea; keonjungkim@gmail.com

**Keywords:** carbon nanotube sheets, elastomer, chemomechanical energy harvesters, wrinkled structure, biaxial, flexible

## Abstract

Mechanical energy harvesters made from soft and flexible materials can be employed as energy sources for wearable and implantable devices. However, considering how human organs and joints expand and bend in many directions, the energy generated in response to a mechanical stimulus in only one direction limits the applicability of mechanical energy harvesters. Here, we report carbon nanotube (CNT) sheets/an elastomer bilayer harvesting electrode (CBHE) that converts two-axis mechanical stimulation into electrical energy. The novel microwinkled structure of the CBHE successfully demonstrates an electrochemical double-layer (EDL) capacitance change from biaxial mechanical stimulation, thereby generating electrical power (0.11 W kg^−1^). Additionally, the low modulus (0.16 MPa) and high deformability due to the elastomeric substrate suggest that the CBHE can be applied to the human body.

## 1. Introduction

Wearable and implantable mechanical energy harvesters have garnered significant attention as a novel power source for self-powered health monitoring, human motion sensing, organ motion tracking, and biomedical uses [1,2,3,4,5,6]. These harvesters have exhibited the potential to supply electrical energy to wearable and implantable electronic devices through human mechanical movements, such as bending, rotating, expanding, and contracting [7,8,9,10,11,12,13,14]. For practical use, mechanical energy harvesters should satisfy two conditions: (i) high deformability to adapt to human movements [15,16] and (ii) matching external mechanical and energy generation stimulation directions.

A chemomechanical energy harvester is an electrochemically based system that includes an energy generation electrode, a counter electrode, and an electrolyte [8,17,18,19]. The energy generation electrode converts external human motion into electrical energy within the electrolytes. This electrode forms an electrochemically accessible area for ions in electrolytes, and mechanical stimuli induce changes in this area to generate electrical energy [18,20]. Therefore, it is necessary to design a soft, flexible, elastic energy generation electrode for human use. Coiled carbon nanotube (CNT) yarns [8,17,18,20,21,22] and those with aligned buckles [14,23,24,25] are representative energy-harvesting electrodes. Leveraging these highly deformable fibrous-type electrodes allows for effective energy harvesting from human muscle, joint, skin, and organ motion. However, these electrodes generate electrical energy that is responsive to only the longitudinal direction of the yarn (i.e., harnessing a uniaxial mechanical stimulus). Given that in reality, humans and organ movements involve multidirectional motion [14,26,27], conventional uniaxial harvesters cannot be applied when the direction of the stimulus does not align with the harvester’s energy generation. Therefore, for practical applications, it is essential to design a chemo-mechanical energy-harvesting electrode capable of accepting mechanical stimulation from multiple directions.

In this study, we investigated a CBHE capable of converting biaxial mechanical energy into electricity for the first time. The microwrinkled structure of the CNT sheets forms due to a strain mismatch between the pre-stretched (80%) elastomer substrate and the CNT sheets, which are distributed evenly on the surface of the CBHE. In addition to the softness, elasticity, and flexibility of the elastomer substrate, the relatively low modulus of the CBHE (0.16 MPa) means that it can be used as a wearable and implantable energy harvester for a human. Stretching at 80% along both the longitudinal and transverse directions enables the unfolding of microwrinkles, which changes the electrochemical capacitance. The CBHE-based chemo-mechanical energy harvesters generate changes at an open-circuit voltage (OCV) of 4 mV and a peak-to-peak short-circuit current (SCC) of 17 µA kg^−1^, when stretching at 80% along both directions at 1 Hz within 0.1 M HCl. Consequently, the CBHE-based chemo-mechanical energy harvesters successfully generate 0.11 W kg^−1^ of electrical power using biaxial stretching motion.

## 2. Materials and Methods

### 2.1. Materials for CNT Sheets/Elastomer Bilayer

The CNT sheets were fabricated from the continuous drawing of a spinnable CNT forest (A-tech system, Incheon, Republic of Korea). The spinnable CNT forest was synthesized using the chemical vapor deposition method at 680 °C using acetylene (C_2_H_2_) gas and silicone wafers coated with an iron catalyst [28,29]. The elastomer layer (SMOOTH-ON, Macungie, PA, USA) was produced by mixing 0.5 mL of silicone resin and curing agent and then casting it in a Petri dish and curing it for 6 h. The CNT sheets/elastomer bilayer was fabricated by stacking the CNT sheets on pre-stretched silicone rubber. Pt wire was used as a current collector, and HCl solution was employed as an electrolyte. Both were purchased from Sigma-Aldrich, Seoul, Republic of Korea.

### 2.2. Material Characterization

The mechanical and electrical stability of the CNT sheets/elastomer bilayer were evaluated using Micro-UTM (CM-Mutm-VT-0030, HEAD, Seoul, Republic of Korea) and a digital multimeter (U1282 handheld digital multimeter, Keysight Technologies, Springs, CO, USA), respectively. A field-emission scanning microscope (FESEM, Hitachi, Tokyo, Japan) was used for the structural analysis of the surface and cross-section of the CNT sheets/elastomer bilayer.

### 2.3. Characterization of Harvesting Performance for CBHE

An electrochemical analysis device (Zive SM6, WonA Tech., Seoul, Republic of Korea) was used for evaluating the harvesting performance. For electrochemical characterization, a three-electrode system was used, which included a reference, a counter, and the CNT sheets/elastomer energy harvester. We plotted cyclic voltammetry (CV) curves to obtain the b-value, the potential of zero charge (PZC), and the capacitance changes in the CBHE. Gravimetric capacitance (C) was calculated as follows:

$$C=\frac{A}{mv∆V}$$

where A represents the area of the CV curve, m denotes the mass of the CNT sheets, v indicates the scan rate, and ∆V stands for the scan range. The real-time OCV and SCC were measured using open-circuit potential measurement and potentiostatic methods, respectively while stretching the CNT sheets/elastomer bilayer to 80% at 1 Hz. Gravimetric peak power (P) was measured in the two-electrode system, including the counter and the CBHE, which was calculated as follows:

$$P=\frac{V^{2}}{R}$$

where *V* represents the generated peak voltage when stretching the CNT sheets/elastomer bilayer, and *R* denotes the load resistance. We conducted all the electrochemical analyses within 0.1 M HCl electrolytes.

## 3. Results and Discussion

**Figure 1:**
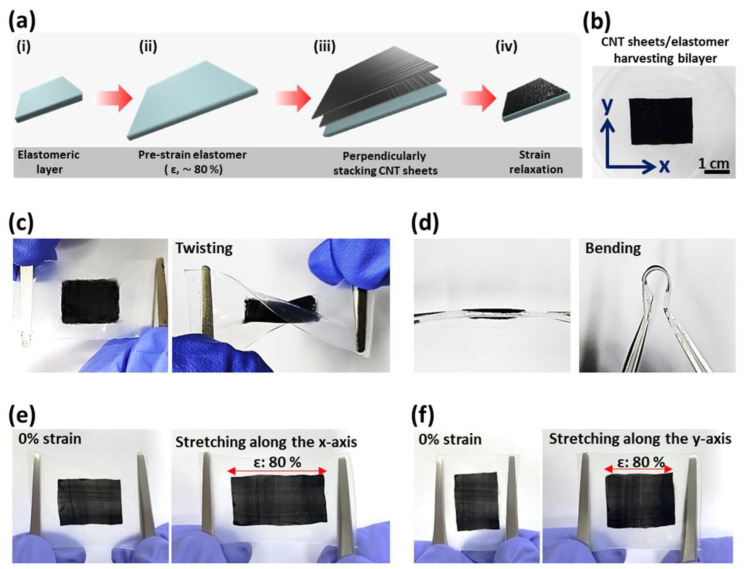
**Fabrication process and stretchability of CBHE:** (**a**) illustration of the process for fabricating CBHE: (**a**)-(i) elastomer, (**a**)-(ii) omnidirectionally pre-stretched elastomer at 80 %, (**a**)-(iii) perpendicularly stacked CNT sheets on the pre-stretched elastomer, and (**a**)-(iv) released CNT sheets stacked on elastomer at 0% (The red arrow indicates the next step of progress). (**b**) Optical image of CBHE (scale bar: 1 cm). Optical images of CBHE before and after (**c**) twisting and (**d**) bending. Optical images of CBHE before and after stretching along (**e**) x-axis and (**f**) y-axis at 80%.

The elastomeric layer was manufactured by uniformly mixing silicone resin and a curing agent in equal proportions and drying the combination at 25 °C for 6 h. The highly stretchy elastomer layer with a height of 650 µm (Figure 1a-(i)) was homogeneously pre-stretched at a rate of 2 mm s^−1^ up to 80% in all directions, as shown in Figure 1a-(ii). The CNT sheets with a 3.6 cm width were continuously drawn from the spinnable CNT forest (see the Section 2). The six-layered CNT sheets were sequentially laminated at right angles starting from the x-axis direction on a pre-stretched elastomer substrate along the stretching direction (Figure 1a-(iii)). The pre-stretched CNT sheets/elastomer was contracted at a rate of 2 mm s^−1^ using the recovery force of the elastomer substrate without structural hysteresis (Figure 1a-(iv)). As shown in Figure 1a-(iii), (iv), the area of the CNT sheets decreased by a factor of 3.24, indicating that the strain relaxation densely packed CNT sheets and stored an extra area. The CNT sheets on the elastomer substrate comprise a random, wrinkled structure due to the strain mismatch between the CNT sheets and the elastomeric substrate [23,24,30]. Figure 1b shows an optical image of the manufactured rectangular-shaped CBHE (2.5 cm × 2 cm). Due to the high elasticity and softness of the elastomeric layer, the CBHE can twist, bend, and stretch, as shown in Figure 1c–f. Despite harsh mechanical deformation, the CBHE exhibits structural stability during stretching and releasing along the x- and y-axes without the CNT sheets detaching from the elastomer layer, as shown in Figure 1e,f.

**Figure 2:**
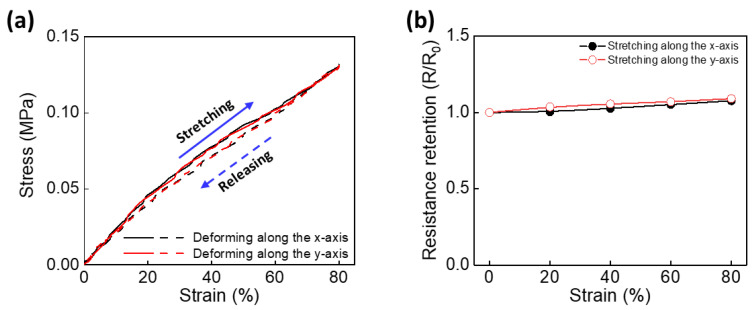
The mechanical and electrical properties of the CBHE: (**a**) the strain–stress graph for the CBHE when stretching along the x-axis (black) and y-axis (red); (**b**) strain versus resistance retention when stretching the CBHE along the x-axis (black solid circle) and y-axis (red open circle).

To confirm the mechanical properties of the CBHE, the strain–stress relationships were measured at a rate of 2 mm s^−1^ after 20 cycles of mechanical training. The 1 cm × 1 cm CBHE consistently required 0.13 MPa to stretch to 80% along both the x- and y-axes. The stress difference rate (i.e., the difference in stress between the x- and y-axes divided by the tensile stress along the x-axis) showed a negligible difference of 2.1% over the entire strain range, indicating analogous mechanical behavior in the CBHE for each axis (Figure 2a). Furthermore, mechanical reversibility and stability were demonstrated using similar strain–stress curves during stretching and releasing after mechanical training (Figure S1). These results indicate that the elastomeric substrate effectively served as a mechanical support with a homogenous structure. Compared to the previously reported elastomer-based chemo-mechanical energy-harvesting electrodes, this CBHE has a remarkably lower modulus of 0.16 MPa when it stretches to 80%. This modulus is relatively lower than that of human soft tissue and organs, such as the muscles, skin, and joints, indicating that the CBHE can be used as a wearable and implantable energy-harvesting electrode [24].

Electrical stability was evaluated at every 20% tensile strain value after 20 cycles of mechanical training (Figure 2b). The length-normalized resistance—resistance divided by the initial length—was 834 Ω cm^−1^ and 844 Ω cm^−1^ before stretching along the x- and y-axes, respectively. After stretching to 80% along the x- and y-axes, resistance retention, which reflects the resistance to tension divided by resistance at 0% strain, was 1.08 and 1.09, respectively. These negligible differences demonstrate that the CBHE maintains electrical stability under mechanical deformation.

**Figure 3:**
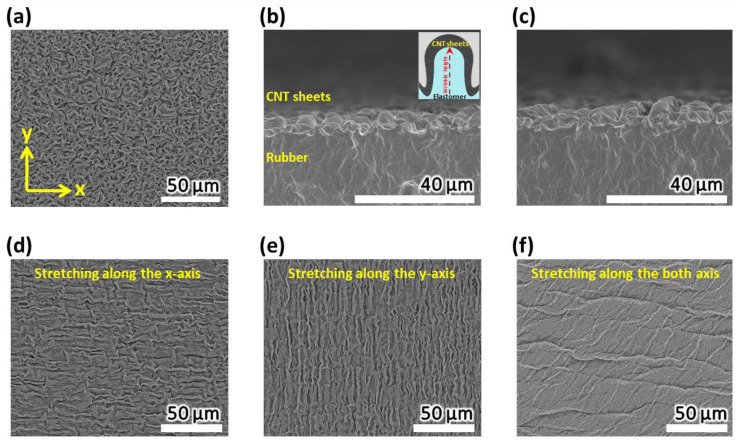
Surface structure analysis: (**a**–**c**) SEM images for CBHE: (**a**) surface SEM images (scale bar: 50 µm) and cross-sectional SEM images along (**b**) x-axis and (**c**) y-axis (scale bar: 40 µm). SEM images for CBHE stretching along (**d**) x-axis, (**e**) y-axis, and (**f**) both axes (scale bar: 50 µm).

The microwrinkled structure of the CNT sheets induced by the strain mismatch between the CNT sheets and the elastomer plays a significant role in chemo-mechanical energy harvesting from mechanical deformation. Owing to the sequential stacking of the CNT sheets, the CBHE achieved a homogeneously distributed, wrinkled structure, as shown in Figure 3a. When observing the CBHE cross-section, about 3 µm high wrinkles were confirmed along the x- and y-axes, as shown in the cross-section scanning electron microscope (SEM) images in Figure 3b,c.

Mechanical stretching changes the surface area stored in the microwrinkles, thereby inducing an EDL capacitance change, which generates electrical energy. Thus, it is essential to confirm the variation in microwrinkles by stretching. Stretching the CBHE removes the microwrinkles perpendicular to the stretching direction, while the microwrinkles parallel to the stretching direction remain. As shown in Figure 3d, the microwrinkles are visible along the x-axis, while the microwrinkles along the y-axis are collapsed. Contrarily, Figure 3d,e shows the microwrinkled structure aligned along the y-axis when stretched. The independent response of these perpendicular microwrinkles suggests that there is no interference between each microwrinkle. Therefore, only the wrinkles oriented perpendicular to the tensile direction contribute to the energy-harvesting performance. Unlike the previously reported unidirectionally aligned buckle structures, the novel, randomly distributed microwrinkle structures effectively accommodate surface area changes in response to biaxial mechanical motion.

Furthermore, this structure was straightened when stretching along both axes, as shown in Figure 3f. The reversible unfolding and folding of the microstructures in the CBHE induced by stretching and releasing indicate reversible variations in the surface area of the CNT sheets. Considering the direction of the stretching-induced surface area change, the CBHE-based chemo-mechanical energy harvester harnesses the biaxial mechanical movement occurring in human joints and organs.

**Figure 4:**
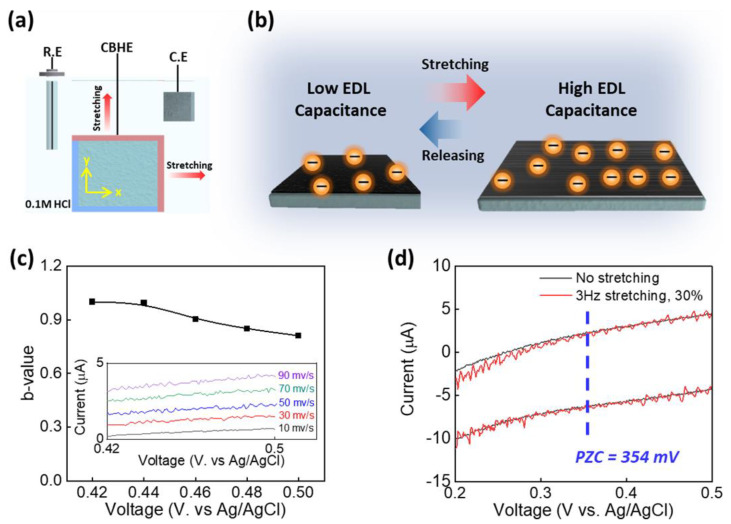
The electrical energy generation mechanism for the CBHE: (**a**) the configuration of the CBHE-based three-electrode system (reference electrode: Ag/AgCl; counter electrode: Pt mesh/CNT bucky paper); (**b**) a conceptual illustration showing the changes in EDL capacitance when stretching and releasing the CBHE; (**c**) the b-value is a function of voltage (inset showing the linear sweep voltammetry for calculating b-value); (**d**) PECS analysis using cyclic voltammetry with (black) and without (red) stretching the CBHE.

To clarify the energy-harvesting mechanism of the CBHE-based chemo-mechanical energy harvesters shown in Figure 4a, a three-electrode system comprising a reference, a counter electrode, and the CBHE was prepared by immersing them within 0.1 M HCl. Figure 4b shows the expected harvesting mechanism for the CBHE-based energy harvester. Before stretching the CBHE, the microwrinkled structure is folded, indicating a decreased ion-accessible area. The stretching-induced unfolding of microwrinkles provides an enhanced ion-accessible area. The variation in the ion-accessible area increases the EDL capacitance in the CBHE [23,24,30], thereby generating a voltage of Q=CV when Q is constant, where the Q represents the charge density, C denotes the capacitance, and V stands for the voltage [8,17,21].

Before characterizing the harvesting performance of the CBHE, it is essential to unveil (1) the ion’s physical adsorption on the CBHE and (2) the polarity of the absorbed ions. The b-value measurement reveals the ions’ physical adsorption as the dominant charge storage mechanism for the CBHE (Figure 4c). The charge storage mechanism is divided into capacitive and diffusion-controlled effects, as in the equation $i=C_{1}v+C_{2}v^{1/2}=av^{b}$, where i represents the current; v stands for the scan rate; and $C_{1}v$ and $C_{2}v^{1/2}$ denote the non-faradic capacitive current and faradic diffusion-controlled current, respectively [31,32]. The b-value for the CBHE is a function of voltage, as shown in Figure 4c. Linear sweep voltammetry within 0.1 M HCl was conducted in the voltage range of 0.42 V to 0.5 V, including the generated voltage range in the CBHE, as shown in the inset of Figure 4c. A b-value in the range between 0.7≤b≤1 and 0.35≤b<0.7 indicates capacitive and diffusion-controlled effects as the governing charge storage mechanism. The CBHE exhibited a b-value ranging from 0.81 to 0.99. The CBHE demonstrated the capacitive effect as the dominant charge storage mechanism as per the b-value, determining the ions’ physical adsorption. Furthermore, the intrinsic bias voltage (OCV at 0% strain−PZC) was analyzed to determine the polarity of the absorbed ions, which is harnessed for harvesting electrical energy. Positive and negative intrinsic bias voltages indicate hole and electron injection in the CBHE from electrolytes, respectively. In the case of hole injection in the CBHE, negative ions are dominantly absorbed at the surface of the CBHE, while positive ions are absorbed at the surface of the CBHE with electron injection [22,33]. PZC determination was accomplished using piezoelectrochemical spectroscopy (PECS) (Figure 4d), which involves a comparison of CV measurements in either static state or repetitive mechanical deformation [17,19]. The PZC was identified in the voltage corresponding to the minimized current. Thus, the PZC for the CBHE was 354, and the intrinsic bias voltage for CBHE was 78 mV (the OCV at 0% in the CBHE was 432 mV). This result indicated that the hole was injected from the electrolytes to the CBHE due to their electrochemical potential differences. Consequently, the CBHE harnessed negative ions to generate electrical energy within 0.1 M HCl.

**Figure 5:**
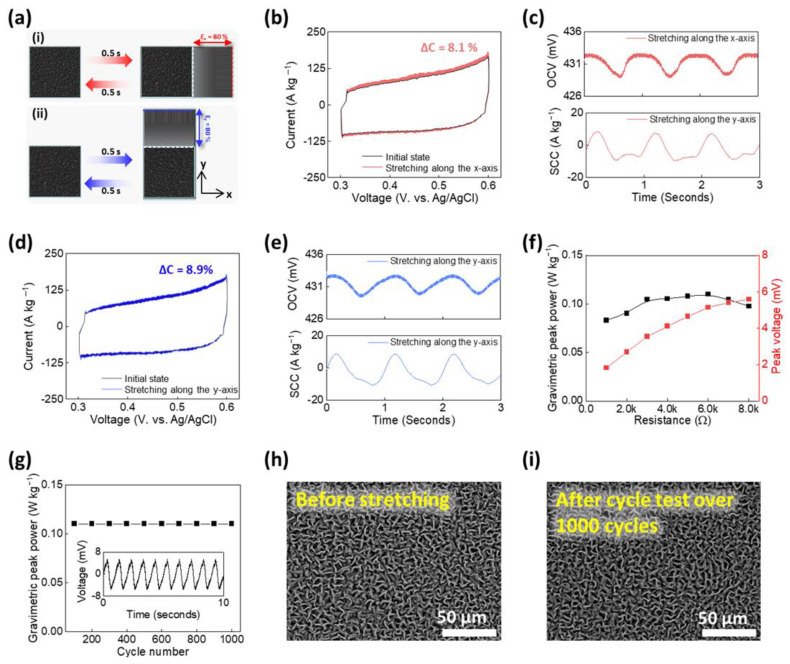
Evaluating harvesting performance within 0.1 M HCl: (**a**) schematic illustrations of harvesting condition: (**a**)-(i) stretching CBHE along the x-axis, (**a**)-(ii) stretching CBHE along the y-axis; (**b**) CV curves before and after stretching CBHE along the x-axis; (**c**) real-time OCV and SCC graphs when sinusoidally stretching CBHE to 80% at 1 Hz along the x-axis; (**d**) CV curves before and after stretching CBHE along the y-axis; (**e**) real-time OCV and SCC graphs when sinusoidally stretching CBHE to 80% at 1 Hz along the y-axis; (**f**) gravimetric peak power and peak voltage of CBHE with load resistance; (**g**) gravimetric peak power of CBHE-based chemo-mechanical energy harvesters during 1000 cycles, when stretched to 80% at 1 Hz (inset showing the generated voltage on 10 cycles). SEM images of CBHE (**h**) before and (**i**) after the harvesting cycle test.

Figure 5a shows the stretching conditions to demonstrate energy harvesting for the CBHE within 0.1 M HCl. The CBHE was sinusoidally stretched to 80% along the x-axis (Figure 5a-(i)) and y-axis (Figure 5a-(ii)) at 1 Hz. To confirm electrical energy generation from the capacitance changes, the gravimetric capacitance of the CBHE was measured before and after being stretched to 80% along the x- and y-axes. When the CBHE was stretched to 80% along the x-axis, the gravimetric capacitance increased by 8.1% from 3.72 F g^−1^ to 3.42 F g^−1^ (Figure 5b). From the stretching-induced changes in capacitance, the CBHE underwent a variation in the OCV of 4 mV and the peak-to-peak SCC of 15.4 µA kg^−1^ when sinusoidally stretched to 80% at 1 Hz (Figure 5c). Similar to the x-axis stretching performance, the CBHE provided a stretching-induced capacitance change of 8.9% from 3.70 F g^−1^ to 3.37 F g^−1^ when stretched to 80% along the y-axis (Figure 5d). Due to the similar stretching-induced capacitance change (Figure 5b,d) induced by the variation in the microwrinkled structure, the CBHE-based chemo-mechanical energy harvester generated a gravimetric peak power of 0.11 W kg^−1^ at 6 kΩ regardless of the stretching direction (Figure 5f). Furthermore, the gravimetric peak power maintained its performance over 1000 cycles (Figure 5g), indicating the reversibility of the microwrinkled structure (Figure 5h,i). Consequently, our CBHE demonstrated energy harvesting via harnessing biaxial mechanical strain for the first time.

## 4. Conclusions

In summary, we developed a CBHE that generates electrical energy using biaxial stimulation for the first time. The highly deformable CBHE provided a remarkably low modulus of 0.16 MPa when stretched to 80% and deformability, such as twisting, bending, and stretching. The novel microwrinkled structure of the elastomer substrate showed ion physical adsorption as the dominant charge storage mechanism, thus demonstrating the applicability of the electrode in chemo-mechanical energy harvesters. Furthermore, the variation in the surface area of the microwrinkles from stretching along the x- and y-axes provided variations in the EDL capacitance (x-axis: 8.1%; y-axis: 8.9%) attributable to biaxial harvesting. Consequently, the CBHE successfully generated a gravimetric peak power of 0.11 W kg^−1^ when stretched along both axes to 80% at 1 Hz.

## Data Availability

The data that support the findings of this study are available upon request from the corresponding author.

## Supplementary Materials

The following supporting information can be downloaded at: https://www.mdpi.com/article/10.3390/polym16172477/s1, Figure S1: Strain–stress curves when stretching CBHE along the (a) x-axis and (b) y-axis during 20 cycles at a rate of 2 mm s^−1^. The black and red show the 1st and 20th cycles, respectively. The solid and dashed lines show the stretching and releasing, respectively.
