# Supplementary material for: Carbon Nanotube Sheets/Elastomer Bilayer Harvesting Electrode with Biaxially Generated Electrical Energy
Source: Polymers (Basel). 2024 Aug 30;16(17):2477. doi: 10.3390/polym16172477 (PMC11398110; doi:10.3390/polym16172477)
Supplement: Supplementary file 1 [file polymers-16-02477-s001.zip › polymers-3158178-supplementary.pdf]

Supplementary material

Carbon nanotube sheets/Elastomer Bilayer Harvesting Electrode with Bi-axially Generating Electrical Energy

Seongjae Oh[1], Hyeon Ji Kim[1], Seon Lee[1], Keon Jung Kim[2] Shi Hyeong Kim[1*]

[1]*Department of Advanced Textile R&D, Korea Institute of Industrial Technology, Ansan 15588, Republic of Korea*

[2]*Semiconductor R&D Center, Samsung Electronics, Hwaseong 18448, Republic of Korea*

*Correspondence and requests for materials should be addressed to S. H. K (shk@kitech.re.kr)

Keywords: Carbon nanotube sheets, Elastomer, Chemo-mechanical energy harvesters, Wrinkled structure, Biaxial, Flexible

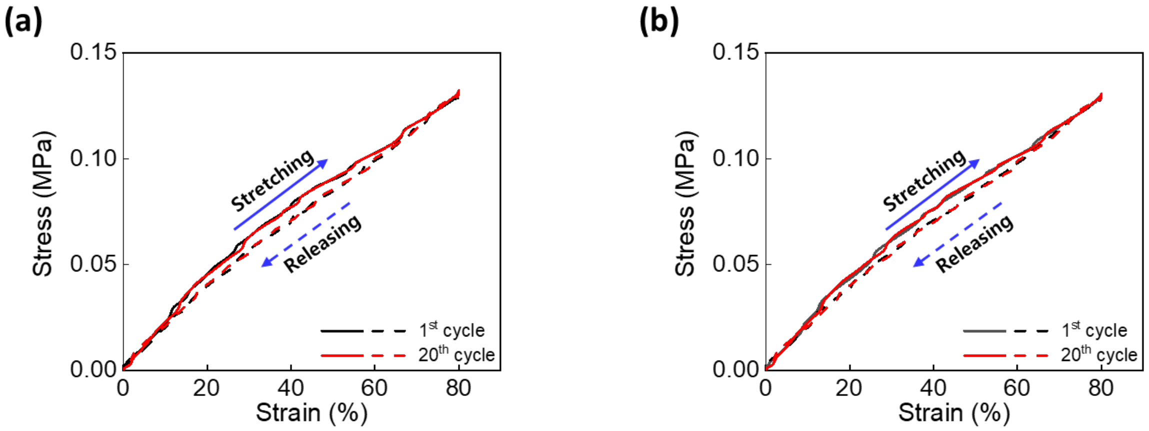

Figure S1. Strain–stress curves when stretching CBHE along the **(a)** x-axis and **(b)** y-axis during 20 cycles at a rate of 2 mm s^{-1}. The black and red show the 1st and 20th cycles, respectively. The solid and dashed lines show the stretching and releasing, respectively.
